# Prognostic value of the ratio of globally sclerotic glomeruli in patients with idiopathic IgA nephropathy

**DOI:** 10.1038/s41598-025-33114-3

**Published:** 2026-01-07

**Authors:** Sinan Kazan, Savaş Öztürk, Müge Uzerk Kibar, Seyda Gul Ozcan, Raife Dilhan Alcelik Karacan, Necmi Eren, Mahmut Gok, Kultigin Turkmen, Hamad Dheir, Taner Basturk, Erhan Tatar, Omer Faruk Akcay, Meltem Gürsu, Hakki Arikan, Sena Ulu, Mehmet Deniz Ayli, Ilhan Kurultak, Dilek Guven Taymez, Belda Dursun, Ramazan Ozturk, Sim Kutlay, Sebnem Karakan, Murvet Yilmaz, Dilek Torun, Kenan Turgutalp, Alper Azak, Zeki Aydin, Deren Oygar, Nedim Selcuk Yilmaz, Bulent Kaya, Zülfükar Yilmaz, Ozcan Uzun, Murat Hayri Sipahioğlu, Melike Betül Öğütmen, Serap Yadigar, Aysegul Oruc, Mahmud Islam, Müge Doksan, Meryem Keles, Mehmet Riza Altiparmak, Abdulkadir Celik, Erkan Dervişoğlu, Rabia Hacer, Ezgi Coskun Yenigun, Onur Tunca

**Affiliations:** 1 Department of Nephrology, Faculty of Medicine, Afyonkarahisar Health Sciences University, Afyonkarahisar, Turkey; 2 Department of Nephrology, Istanbul Faculty of Medicine, Istanbul University, Istanbul, Turkey; 3 Clinic of Nephrology, Ankara Bilkent City Hospital, Ankara, Turkey; 4 Division of Nephrology, Department of Internal Medicine, Cerrahpasa Faculty of Medicine, Istanbul University, Istanbul, Turkey; 5 Division of Nephrology, Department of Internal Medicine, Haseki Training and Research Hospital, Istanbul, Turkey; 6 Department of Nephrology, Faculty of Medicine, Kocaeli University, İzmit, Turkey; 7 Department of Nephrology, Sultan 2. Abdulhamid Han Training and Research Hospital, University of Health Sciences, Istanbul, Turkey; 8 Department of Nephrology, Faculty of Medicine, Necmettin Erbakan University, Konya, Turkey; 9 Department of Nephrology, Faculty of Medicine, Sakarya University, Sakarya, Turkey; 10 Department of Nephrology, Hamidiye Etfal Training and Research Hospital, University of Health Sciences, Istanbul, Turkey; 11 Medical Point Hospital, Clinic of Nephrology, Izmir Economy University, İzmir, Turkey; 12 Department of Nephrology, Faculty of Medicine, Gazi University, Ankara, Turkey; 13 Department of Nephrology, Faculty of Medicine, Bezmialem Vakif University, Istanbul, Turkey; 14 Department of Nephrology, School of Medicine, Marmara University, Istanbul, Turkey; 15 Department of Nephrology, Etlik City Hospital, University of Health Sciences, Ankara, Turkey; 16 Department of Nephrology, Faculty of Medicine, Trakya University, Edirne, Turkey; 17 Clinic of Nephrology, Kocaeli State Hospital, İzmit, Turkey; 18 Department of Nephrology, Faculty of Medicine, Pamukkale University, Denizli, Turkey; 19 Department of Nephrology, Ankara Training and Research Hospital, Ankara, Turkey; 20 Department of Nephrology, Faculty of Medicine, Ankara University, Ankara, Turkey; 21 Department of Nephrology, Faculty of Medicine, Ankara Yildirim Beyazit University, Ankara, Turkey; 22 Department of Nephrology, Bakirkoy Dr. Sadi Konuk Training and Research Hospital, University of Health Sciences, Istanbul, Turkey; 23 Department of Nephrology, Faculty of Medicine, Dr. Turgut Noyan Medical and Research Center, Baskent University, Adana, Turkey; 24 Division of Nephrology, Department of Internal Medicine, Faculty of Medicine, Mersin University, Mersin, Turkey; 25 Department of Nephrology, Balikesir Ataturk Education and Research City Hospital, University of Health Sciences, Balikesir, Turkey; 26 Department of Nephrology, Darica Farabi Training and Research Hospital, University of Health Sciences, İzmit, Turkey; 27 Division of Nephrology, Dr. Burhan Nalbantoglu State Hospital, Lefkosa, Cyprus; 28 Department of Nephrology, Faculty of Medicine, Cukurova University, Adana, Turkey; 29 Department of Nephrology, Faculty of Medicine, Dicle University, Diyarbakir, Turkey; 30 Department of Nephrology, Faculty of Medicine, Erciyes University, Kayseri, Turkey; 31 Department of Nephrology, Haydarpasa Numune Training and Research Hospital, University of Health Sciences, Istanbul, Turkey; 32 Clinic of Nephrology, Kartal Dr. Lutfi Kirdar City Hospital, Istanbul, Turkey; 33 Department of Nephrology, Faculty of Medicine, Bursa Uludag University, Bursa, Turkey

**Keywords:** Glomerulosclerosis, IgA nephropathy, Kidney outcome, Prognostic index, Diseases, Medical research, Nephrology

## Abstract

IgA nephropathy (IgAN) is the most common primary glomerulonephritis worldwide. We assessed whether the Ratio of Globally Sclerotic Glomeruli (RoGSG) on diagnostic biopsy predicts subsequent kidney outcomes in a nationwide, multi‑center registry. Among 326 adults with idiopathic IgAN (mean age 39.1 ± 12.8 years; 60.1% male), 43 patients (13.2%) met a 5 year composite outcome defined as any of: doubling of serum creatinine or ≥ 50% decline in eGFR from baseline, eGFR < 15 mL/min/1.73 m^2^, or initiation of kidney replacement therapy. Receiver operating characteristic analysis identified a RoGSG cutoff of 28.86% for predicting the composite outcome (AUC 0.917, 95% CI 0.885–0.949; sensitivity 93.0%; specificity 84.5%). Using this threshold, 47.6% of patients with RoGSG ≥ 28.86% versus 1.2% with RoGSG < 28.86% reached the composite outcome. In multivariable models adjusted for clinical and pathologic covariates, high RoGSG, grade 2 tubular atrophy/interstitial fibrosis, and non‑response to initial immunosuppression were independent predictors of adverse outcomes. The prognostic association of RoGSG persisted in key subgroups, including those with nephrotic syndrome and those with initial treatment response. These findings support RoGSG as a readily available histopathologic marker that may improve risk stratification in IgAN; however, prospective studies and external validation in independent cohorts are required before any clinical adoption.

**Supplementary Information:**

The online version contains supplementary material available at 10.1038/s41598-025-33114-3.

## Introduction

IgA nephropathy (IgAN) is the most common primary glomerulonephritis both in Turkey and worldwide^1,2^. Although it generally exhibits a relatively mild course compared to other glomerular diseases, it can progress to kidney failure (KF) in patients with certain clinical, laboratory, and pathological risk factors. Poor prognostic factors include demographic features such as advanced age, race, and hypertension; laboratory findings such as the presence and severity of proteinuria and low estimated glomerular filtration rate (eGFR) at diagnosis; and pathological features described by the Oxford classification (MEST‑C score), including mesangial hypercellularity, endocapillary hypercellularity, segmental sclerosis, tubular atrophy/interstitial fibrosis, and crescents^3^.

Global glomerulosclerosis is a marker of irreversible kidney damage and reduced kidney reserve. As sclerosis develops in some glomeruli, the functional burden on the remaining intact glomeruli increases, resulting in an elevated single‑nephron GFR^4^. Increased single‑nephron GFR may also lead glomerulosclerosis^5^. Glomerulosclerosis is considered a histopathological finding of chronicity in patients with IgAN and is one of the indicators of progression^6^. The Ratio of Globally Sclerotic Glomeruli (RoGSG) to the total number of glomeruli reflects the extent of irreversible parenchymal injury. This quantitative parameter, although not included in the Oxford classification, may provide additional prognostic information. Therefore, understanding its association with kidney outcomes may contribute to more accurate risk stratification in patients with IgAN.

In this study, we aimed to investigate the prognostic significance of the RoGSG on kidney outcomes in patients with idiopathic IgAN.

## Results

A total of 326 patients with idiopathic IgAN were included. The mean age at diagnosis was 39.1 ± 12.8 years, and 60.1% (n = 196) were male. Hypertension and diabetes mellitus were present in 32.8% and 8.0%, respectively; 16% reported active smoking. Nephrotic syndrome was present in 21.8% (n = 71). Baseline laboratory parameters included median serum creatinine 1.1 mg/dL (IQR 0.8–1.6), BUN 17 mg/dL (IQR 13–25), serum albumin 4.0 g/dL (IQR 3.6–4.3), and spot urine protein‑to‑creatinine ratio 1550 mg/g (IQR 800–3015). Table 1 summarizes baseline features.

**Table 1:** General characteristics of all patients and comparison in terms of composite kidney endpoint status.

		Composite kidney endpoint		
Characteristics	All patients (n = 326)	Present (n = 43)	Absent (n = 283)	*p*
Age at diagnosis, years	39.1 ± 12.8	36.7 ± 12.9	39.4 ± 12.8	0.200
Male gender, %-n	60.1–196	67.4–29	59–157	0.320
Hypertension, %-n	32.8–107	27.9–12	33.6–95	0.492
Diabetes, %-n	8–26	4.7–2	8.5–24	0.551
Active smoker, %-n	16–52	48.8–21	11–31	< 0.001
Nephrotic syndrome, %-n	21.8–71	41.9–18	18.7–53	0.001
Immunosuppression, %-n	56.4–184	65.1–28	55.1–156	0.250
Response to immunosuppression, %-n	86.4–159	39.3–11	94.9–148	< 0.001
BUN at diagnosis, mg/dL	17 (13–25)	23 (16–30)	16 (13–24)	< 0.001
Serum creatinine at diagnosis, mg/dL	1.1 (0.8–1.6)	1.5 (1–1.8)	1.1 (0.8–1.5)	0.002
Serum albumin, g/dL	4 (3.6–4.3)	3.6 (3.3–4)	4 (3.7–4.4)	< 0.001
Hemoglobin, g/dL	13.3 ± 2.1	13.3 ± 1.6	13.3 ± 2.1	0.818
Spot urine protein/creatinine ratio, mg/g	1550 (800–3015)	2100 (1263–3800)	1385 (800–2950)	0.034
Mesangial hypercellularity, %-n M0 M1	22.4–73 77.6–253	14–6 86–37	23.7–67 76.3–216	0.174
Endocapillary hypercellularity, %-n E0 E1	78.5–256 21.5–70	60.5–26 39.5–17	81.3–230 18.7–53	0.004
Segmental sclerosis, %-n S0 S1	56.7–185 43.3–141	58.1–25 41.9–18	56.5–160 43.5–123	0.870
Tubular atrophy/interstitial fibrosis, %-n T0 T1 T2	57.4–187 33.7–110 8.9–29	18.6–8 32.6–14 48.8–21	63.3–179 33.9–96 2.8–8	< 0.001
Crescents, %-n C0 C1 C2	77.6–253 18.7–61 3.7–12	67.4–29 23.3–10 9.3–4	79.2–224 18–51 2.8–8	0.065
Total number of glomeruli	15 (9.8–23)	12 (9–18)	16 (10–23)	0.124
Number of globally sclerotic glomeruli	2 (1–4)	5 (4–8)	2 (0–3)	< 0.001
RoGSG, %	12.5 (3.2–29.6)	40 (35.7–50)	11.1 (0–22.2)	< 0.001

RoGSG, ratio of global sclerotic glomeruli; BUN, blood urea nitrogen.

### Composite renal outcomes

Over 5 years, 43 patients (13.2%) reached the composite outcome. Among them, 62.8% required kidney replacement therapy, 30.2% experienced doubling of serum creatinine, and 7.0% progressed by eGFR < 15 mL/min/1.73 m^2^. Patients who reached the outcome had higher baseline creatinine and BUN, lower albumin, more frequent nephrotic syndrome and active smoking, and lower response rates to immunosuppression (Table 1).

### Predictive value of RoGSG

ROC analysis identified a RoGSG cutoff of 28.86% (AUC 0.917, 95% CI 0.885–0.949; *p* < 0.001; sensitivity 93.0%; specificity 84.5%). When stratified by this threshold, 84 patients (25.8%) were high RoGSG (≥ 28.86%) and 242 (74.2%) were low (< 28.86%). Composite outcome incidence was 47.6% in high versus 1.2% in low RoGSG groups (*p* < 0.001; Table 2).

**Table 2:** Comparison of the patients in terms of RoGSG groups.

	RoGSG Group		
Characteristics	Low RoGSG (< 28.86) (n = 242)	High RoGSG (≥ 28.86) (n = 84)	*p*
Age at diagnosis, years	39.01 ± 13.2	39.18 ± 11.9	0.823
Male gender, %-n	58.7–142	64.3–54	0.438
Hypertension, %-n	30.6–74	39.3–33	0.177
Diabetes, %-n	7.9–19	8.3–7	0.820
Active smoker, %-n	11.6–28	28.6–24	< 0.001
Nephrotic syndrome, %-n	20.2–49	26.2–22	0.283
Immunosuppression, %-n	53.7–130	64.3–54	0.098
Response to immunosuppression, %-n	93.1–121	70.4–38	< 0.001
BUN at diagnosis, mg/dL	16 (12–22)	23 (16–30)	< 0.001
Serum creatinine at diagnosis, mg/dL	1.1 (0.8–1.5)	1.5 (1.1–1.8)	< 0.001
Serum albumin, g/dL	4 (3.7–4.3)	3.9 (3.5–4.2)	0.056
Hemoglobin, g/dL	13.21 ± 2.1	13.41 ± 1.8	0.409
Spot urine protein/creatinine ratio, mg/g	1357 (791–2800)	1785 (1075–3771)	0.030
Mesangial hypercellularity, %-n M0 M1	24.4–59 75.6–183	16.7–14 83.3–70	0.172
Endocapillary hypercellularity, %-n E0 E1	80.2–194 19.8–48	73.8–62 26.2–22	0.222
Segmental sclerosis, %-n S0 S1	58.3–141 41.7–101	52.4–44 47.6–40	0.372
Tubular atrophy/interstitial fibrosis, %-n T0 T1 T2	64–155 32.6–79 3.3–8	38.1–32 36.9–31 25–21	< 0.001
Crescents, %-n C0 C1 C2	80.6–195 17.8–43 1.7–4	69–58 21.4–18 9.5–8	0.002
Composite kidney endpoint, %-n	1.2–3	47.6–40	< 0.001

RoGSG, ratio of global sclerotic glomeruli; BUN, blood urea nitrogen.

### Subgroup analyses

To explore whether the prognostic effect of high RoGSG (≥ 28.86%) varied across clinically relevant strata, we performed exploratory subgroup analyses according to nephrotic syndrome, response to immunosuppression, smoking status, TA/IFTA grade (T0–T1 vs T2), and dichotomized medians of baseline proteinuria, serum creatinine, and age. For each stratum, event frequencies and unadjusted odds ratios (ORs) were calculated for high versus low RoGSG.

Heterogeneity was assessed using logistic regression models containing a multiplicative RoGSG × subgroup interaction term. Given limited events and multiple testing, these analyses were considered hypothesis-generating.

### Regression analyses

In multivariable analysis (Table 3), high RoGSG (≥ 28.86%) and TA/IFTA grade 2 remained independently associated with the composite outcome, whereas other covariates lost statistical significance after adjustment. Non-response to initial immunosuppression was also independently associated with higher risk, consistent with univariate findings.

**Table 3:** Risk factors for composite kidney endpoint.

	Univariate			Multivariate		
Characteristics	OR	95% CI	*p*	OR	95% CI	*p*
Nephrotic syndrome (vs. absent)	3.125	1.590–6.140	< 0.001	0.708	0.043–11.548	0.809
Active smoking (vs non-smoker)	7.760	3.835–15.700	< 0.001	16.324	0.885–301.097	0.060
Response to immunosuppression (vs. resistant)	0.035	0.012–0.099	< 0.001	0.014	0.001–0.376	0.011
BUN (per mg/dL)	1.000	1–1.040	0.056			
Creatinine (per mg/dL)	1.805	1.078–3.021	0.025	0.162	0.009–2.786	0.210
Protein to creatinine ratio (per mg/g)	1.002	1.001–1.005	0.008	0.999	0.999–1.000	0.135
Albumin (per g/dL)	0.317	0.187–0.547	< 0.001	0.126	0.013–1.246	0.076
Endocapillary hypercellularity (vs. E0)	2.837	1.437–5.603	0.003	10.710	0.786–146.026	0.075
Segmental sclerosis (vs. S0)	0.937	0.489–1.794	0.843			
Tubular atrophy/interstitial fibrosis (vs. T0) T1 T2	3.263 58.783	1.322–8.053 19.961–172.822	0.010 < 0.001	2.646 33.915	0.223–31.458 28.181–408.180	0.441 < 0.001
High RoGSG (vs. low RoGSG)	72.424	21.457–244.461	< 0.001	33.182	6.233–179.161	0.004

BUN, blood urea nitrogen; RoGSG, ratio of global sclerotic glomeruli.

## Discussion

In this multicenter retrospective cohort, a higher RoGSG at diagnosis was strongly associated with adverse 5 year kidney outcomes in idiopathic IgAN, independent of clinical and histopathologic covariates. Our findings complement prior reports linking global glomerulosclerosis with progression and extend them by proposing an empirically derived threshold that may aid clinical risk stratification. Although several scoring and glomerular grading systems have been proposed to predict the prognosis of IgAN, each has its own limitations, and histopathological assessment remains a matter of ongoing discussion^7–10^. In this context, our study aimed to evaluate the predictive value and prognostic significance of RoGSG in relation to kidney outcomes.

Previous studies have consistently demonstrated that histopathological alterations in glomerulonephritis are significantly associated with disease chronicity and poor kidney outcomes. Global glomerulosclerosis is also one of these histopathological changes. Nakakita et al. recently conducted a study including patients with IgAN, and found that the percentage of global glomerulosclerosis was an independent risk factor for poor kidney outcomes in glomerulonephritis, regardless of the underlying disease^11^. Worawichawong et al. reported that global glomerulosclerosis, despite not being part of the Oxford classification, was associated with C4d deposition and unfavorable renal outcomes in patients with IgAN^12^. Nasri et al. reported that proportion of globally sclerotic glomeruli in patients with IgAN was significantly associated with various clinical and histopathological parameters^13^. In their multicenter retrospective study, Zou et al. demonstrated that the global glomerulosclerosis rate was a significant predictor of disease progression in IgAN^14^. Tan et al. found that global glomerulosclerosis may be a prognostic marker in patients with IgA vasculitis and nephritis^15^. The rate of global sclerosis has not only been investigated in patients with IgAN but also in other glomerulonephritis. Erdogmus et al., based on their 13 year cohort of patients with crescentic glomerulonephritis, identified that a global glomerulosclerosis rate greater than 24% was significantly associated with progression to ESRD^16^. Fakhrjou et al. demonstrated that in primary focal segmental glomerulosclerosis, the proportion of sclerotic glomeruli showed a strong correlation with serum creatinine levels and the degree of tubulointerstitial fibrosis^17^.

More recently, several contemporary studies have further refined prognostic understanding in IgA nephropathy. In a large long-term cohort, even clinically low-risk patients demonstrated substantial progression to kidney failure, emphasizing the need for refined risk stratification^18^. Jiang et al. showed that patients presenting with nephrotic syndrome had markedly worse renal survival, which aligns with our subgroup findings showing that high RoGSG retained prognostic significance even in this group^19^. Shirai S et al. found that even patients with mild proteinuria in IgAN and seemingly favorable baseline clinical status had significantly increased risk of renal progression when presenting with eGFR < 60 mL/min/1.73 m^2^, older age and higher serum IgA levels. This underscores the notion that sole reliance on conventional risk indicators may underestimate future adverse renal outcomes, reinforcing the potential value of additional histopathologic markers such as the RoGSG ratio in our cohort^3^. Our study adds to this growing body of evidence by examining RoGSG in a cohort of different ethnic background and by exploring a possible cutoff value that may aid in future validation efforts.

However, we caution against interpreting 28.86% as a definitive universal cutoff. The threshold was derived and evaluated in the same dataset and requires internal (e.g., bootstrapping) and external validation in independent cohorts before clinical adoption. Accordingly, we present this work as hypothesis‑generating.

Selection and exclusion bias merit emphasis. TSN‑GOLD is a registry drawing primarily from tertiary centers; referred patients may have had more severe disease than those managed locally. Moreover, the registry lacked complete follow‑up for many IgAN entries; our analytic cohort excluded individuals without adequate data, which could bias effect estimates. We could not reliably quantify loss‑to‑follow‑up (LFU) among non‑included patients; thus, we cannot determine whether LFU was random or selective—an acknowledged limitation.

Biopsy acquisition is not standardized across centers. RoGSG may vary by cortical depth (superficial vs. juxtamedullary sampling) and by biopsy adequacy. Although biopsy adequacy was not pre‑specified in TSN‑GOLD and location metadata were not uniformly captured, these factors could influence RoGSG and should be addressed in future prospective studies.

Our subgroup analyses—particularly among patients with nephrotic syndrome and among initial responders—suggest that RoGSG conveys prognostic information beyond early clinical course. We summarize these interactions qualitatively in Supplementary Table S1 to avoid over‑precision not supported by registry granularity. Future work should test interactions formally in larger datasets with standardized treatments and longitudinal covariates (blood pressure, proteinuria).

Finally, while our multivariable model included multiple pathological features, high RoGSG and grade 2 TA/IF retained independent associations with outcome, supporting additive prognostic value. Still, unmeasured confounding remains possible.

Exploratory subgroup analyses (Supplementary Table S1) revealed directionally consistent associations of high RoGSG with the composite outcome across clinical strata, without statistically significant interaction. This suggests that the prognostic contribution of global glomerulosclerosis is relatively uniform across patient subgroups, although the limited event number warrants cautious interpretation.

Although the study has several strengths, certain aspects should be interpreted with caution. The multicenter design and relatively large, well-characterized cohort enhance the generalizability within the registry context, yet the retrospective nature may have introduced inherent biases in patient selection and data collection. In addition, inter-observer variability among centers and lack of prespecified biopsy adequacy could have influenced the consistency of pathological assessment. Age-related changes in global glomerulosclerosis were not adjusted for, which might have affected interpretation. Because of limitations in data capture, time-to-event and competing-risk analyses could not be performed and this is acknowledged as a key limitation, and analyses relied solely on baseline covariates. The RoGSG threshold was derived and evaluated in the same dataset without internal validation, which may have led to optimistic estimates of discrimination. Future work should include internal validation (e.g., bootstrapping, cross-validation) and external validation in prospective cohorts. Moreover, incomplete longitudinal data on treatment, blood pressure, and proteinuria, as well as heterogeneity in management approaches, leave room for residual confounding. Finally, since this registry reflects data from a single country, extrapolation to other populations should be made cautiously.

In summary, our findings suggest that a higher RoGSG at diagnosis is associated with an increased likelihood of adverse kidney outcomes in patients with idiopathic IgAN. This association appeared consistent across key clinical and treatment subgroups. When considered alongside other pathological markers, RoGSG may offer additional insight into prognosis and risk stratification. However, further studies—especially prospective, multiethnic, and externally validated ones—are needed before RoGSG can be incorporated into formal predictive models or clinical decision frameworks.

## Material and methods

### Patients

This multi‑center study is based on the Turkish Society of Nephrology Primary Glomerular Diseases Registry (TSN‑GOLD) and includes adult patients with biopsy‑proven idiopathic IgAN. To ensure a minimum of 5 years of follow‑up for outcome ascertainment, we included patients who underwent kidney biopsy on or before March 31, 2020 (cutoff chosen to allow complete 5 year follow‑up by March 2025). Patients with eGFR < 15 mL/min/1.73 m^2^ at diagnosis and those lacking adequate pathological or clinical follow‑up data were excluded. A total of 33 institutions contributed cases to this cohort.

### Data definitions and timing of measurements

Demographics (age at diagnosis, sex), comorbidities (hypertension, diabetes mellitus), smoking status, presence of nephrotic syndrome, histopathology (Oxford MEST‑C), initiation and response to initial immunosuppressive therapy, and laboratory parameters were recorded. Baseline laboratory values, including serum albumin and hemoglobin, were defined as the closest measurements to the biopsy date within ± 14 days. Response to immunosuppression was defined as partial or complete remission per registry definitions.

### Handling of missing data

We performed complete‑case analyses without imputation. Variables with > 10% missingness were not included in multivariable models.

### Treatment regimen information

**Fig. 1:**
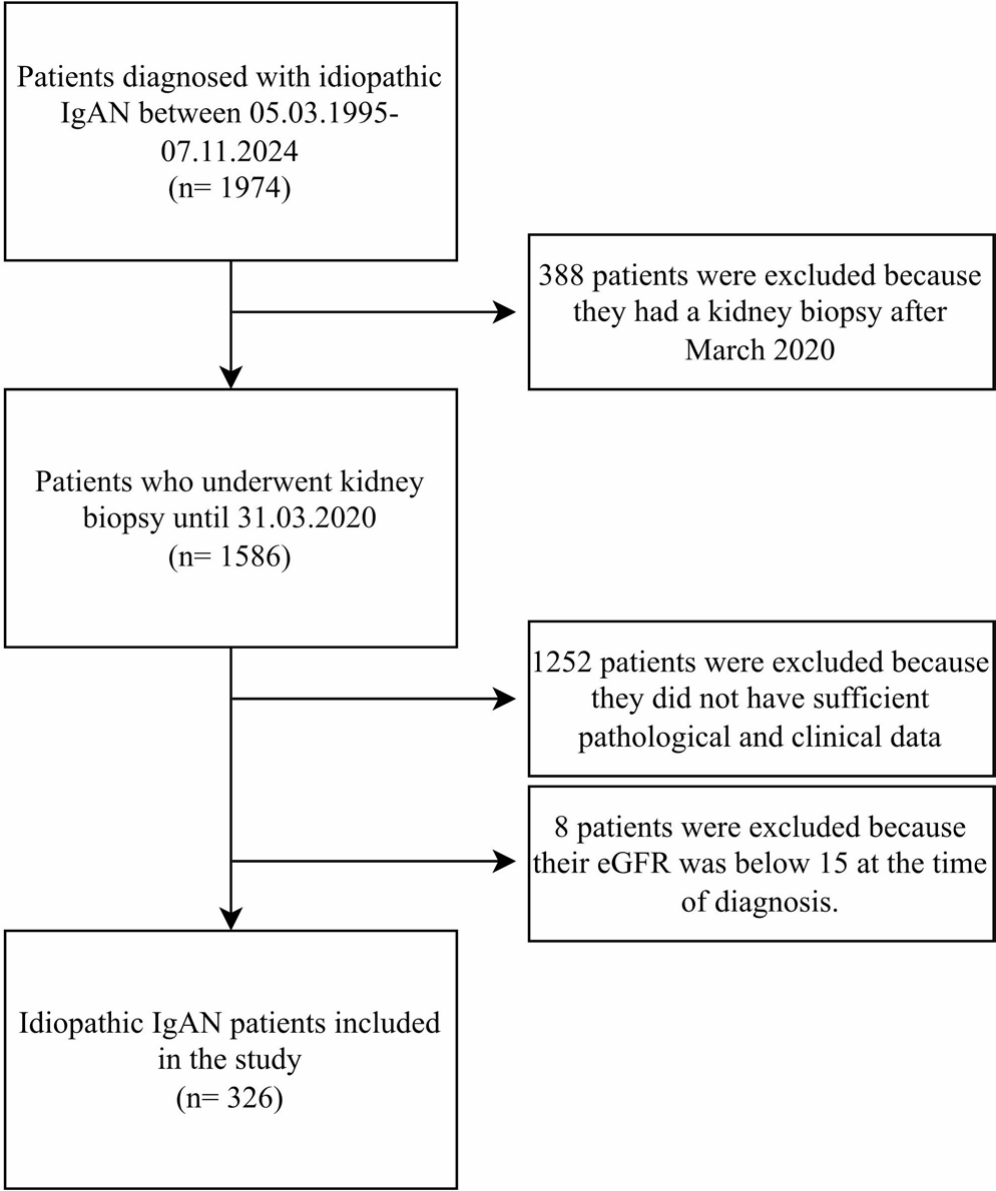
Flowchart of patient selection. Of 1974 IgAN patients in the registry, 326 met eligibility (biopsy on/before 31 March 2020, adequate data, eGFR ≥ 15 mL/min/1.73 m^2^).

**Fig. 2:**
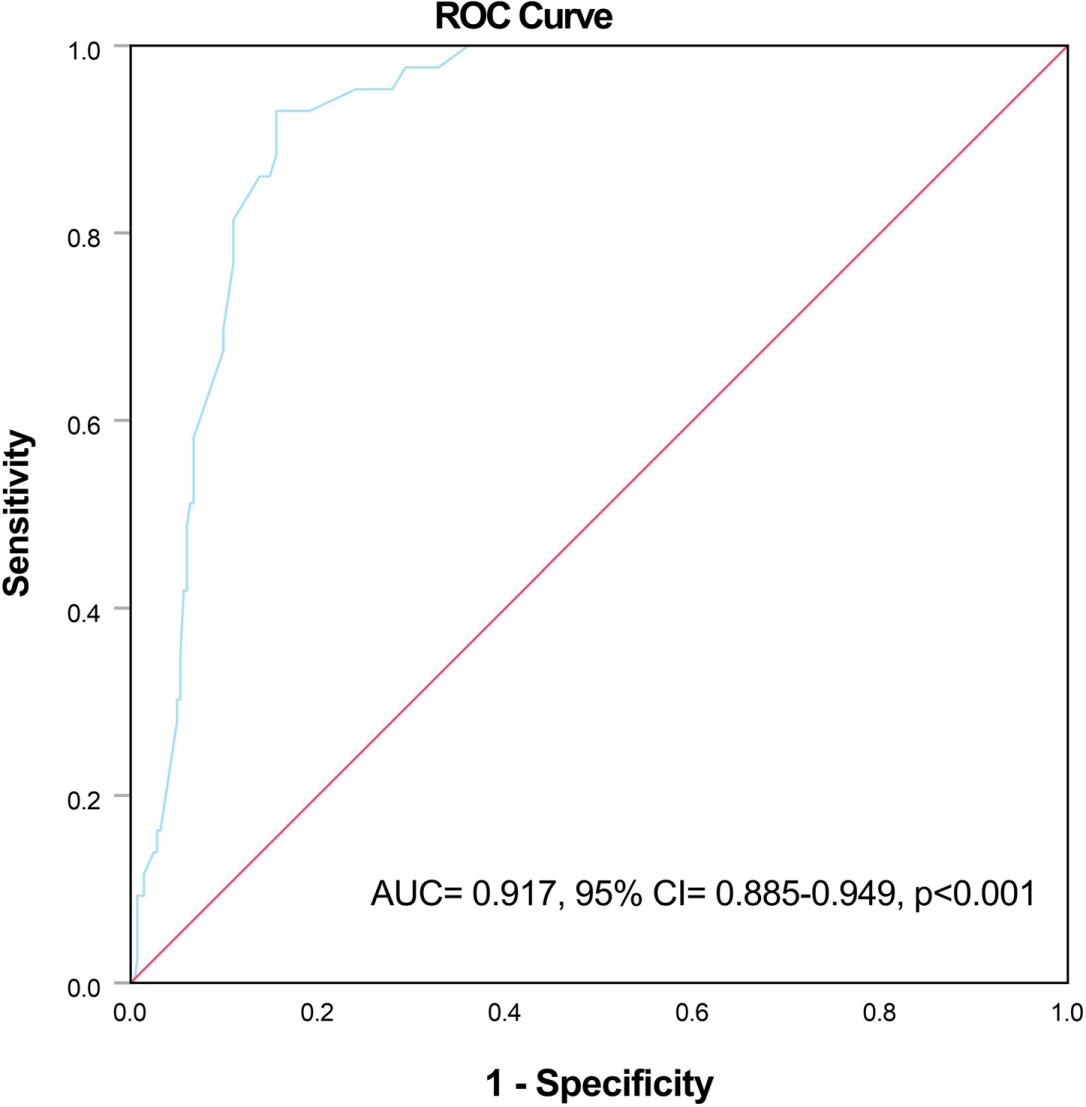
ROC curve of RoGSG predicting the composite kidney outcome (AUC 0.917, 95% CI 0.885–0.949; *p* < 0.001).

Initial immunosuppression was administered in 56.4% (n = 184). Regimens were categorized as corticosteroids alone, corticosteroids plus cyclophosphamide, calcineurin inhibitors, mycophenolate mofetil, or supportive care only. The majority of patients received steroid-based regimens, whereas alternative immunosuppressive agents were used only in a small subset of cases. Agent-level dosing and duration were not consistently available across all centers in the registry; therefore, regimen-level summaries are provided Figs. 1 and 2.

### Outcome

A composite kidney endpoint was considered as the development of any of the following within the 5 year follow-up period:

- Doubling of serum creatinine or a decrease in eGFR of more than 50% from baseline,
- Reduction in eGFR to less than 15 ml/min/1.73 m^2^,
- Initiation of renal replacement therapy (hemodialysis, peritoneal dialysis, or undergoing kidney transplantation).

### RoGSG

The RoGSG was calculated as the percentage of globally sclerotic glomeruli, using the formula: (Number of global sclerotic glomeruli / Number of total glomeruli) × 100.

### Ethics approval

All methods were carried out in accordance with relevant guidelines and regulations. The study protocol was reviewed and approved by the Istanbul University Istanbul Faculty of Medicine Clinical Research Ethics Committee at their meeting dated 29.06.2011 with decision number 09. As this was a retrospective analysis of anonymized data from the TSN-GOLD registry, the requirement for informed consent was waived by the ethics committee.

### Statistical analyses

All statistical analyses were performed using SPSS version 27.0.1 (IBM Corp., Armonk, NY, USA). Two-sided *p* < 0.05 considered significant. Categorical variables were summarized as frequencies and percentages. Continuous variables were assessed for normality using the Shapiro–Wilk test and visual inspection of histograms. Normally distributed continuous variables were expressed as mean ± standard deviation (SD), while non-normally distributed variables were reported as median with interquartile range (Q1-Q3).

Patients were divided into two groups according to kidney outcomes: those who developed the composite endpoint and those who did not. Comparisons between groups were made using the chi-square test for categorical variables. The Fisher’s exact test was used in cases where chi-square test requirements were not met. For continuous variables, the independent samples t-test was used when the assumption of normality was met, and the Mann–Whitney U test was applied for non-normally distributed data.

The optimal cutoff value of the RoGSG in predicting the composite kidney outcome was determined using receiver operating characteristic (ROC) curve analysis. The Youden index was used to identify the best threshold. Patients were then reclassified into two groups based on this cutoff value.

Characteristics that were found to differ between patients with and without a composite kidney endpoint were considered as possible risk factors and included in logistic regression analysis. Variables associated with the composite kidney endpoint in univariate analysis were entered into a multivariate logistic regression model to assess their potential as independent risk factors. The enter method was used for multivariate analyses.

## Supplementary Information

Below is the link to the electronic supplementary material.

Supplementary Material 1

## Data Availability

The data that support the findings of this study were obtained from the Turkish Society of Nephrology Primary Glomerular Diseases Registry (TSN-GOLD). This registry is not publicly available and can only be accessed by authorized researchers with an institutional username and password. Data are available from the Turkish Society of Nephrology upon reasonable request and with appropriate institutional and ethical approvals. Requests for data Access should be directed to the corresponding author (Dr. Sinan Kazan, e-mail: [sinankazan@hotmail.com] (mailto:sinankazan@hotmail.com)).
