# Supplementary material for: Prognostic value of the ratio of globally sclerotic glomeruli in patients with idiopathic IgA nephropathy
Source: Sci Rep. 2026 Jan 7;16:3178. doi: 10.1038/s41598-025-33114-3 (PMC12830591; doi:10.1038/s41598-025-33114-3)
Supplement: Supplementary file 1 — Supplementary Material 1 [file 41598_2025_33114_MOESM1_ESM.docx]

**Supplementary Table S1. Subgroup analyses of high vs. low RoGSG across key clinical strata**

| **Subgroup** | **High RoGSG (n/events)** | **Event rate (%)** | **Low RoGSG (n/events)** | **Event rate (%)** | **Unadjusted OR (95% CI)** | **p value** | **Interaction p** |
| --- | --- | --- | --- | --- | --- | --- | --- |
| **Nephrotic syndrome** | 22 / 10 | 45.5 | 49 / 8 | 16.3 | 4.32 (1.45–12.83) | 0.009 | 0.42 |
| **No nephrotic syndrome** | 62 / 30 | 48.4 | 193 / 3 | 1.6 | 59.8 (17.4–205.7) | <0.001 |  |
| **Response to IS present** | 38 / 8 | 21.1 | 121 / 2 | 1.7 | 14.9 (2.9–75.2) | 0.001 | 0.36 |
| **Response to IS absent** | 46 / 32 | 69.6 | 38 / 9 | 23.7 | 7.79 (3.2–19.0) | <0.001 |  |
| **Active smoker** | 24 / 12 | 50.0 | 28 / 3 | 10.7 | 8.33 (2.0–34.9) | 0.004 | 0.28 |
| **Non-smoker** | 60 / 28 | 46.7 | 214 / 5 | 2.3 | 37.5 (14.6–96.4) | <0.001 |  |
| **TA/IFTA grade T2** | 21 / 10 | 47.6 | 8 / 2 | 25.0 | 2.71 (0.47–15.5) | 0.26 | 0.33 |
| **TA/IFTA grade T0–T1** | 63 / 30 | 47.6 | 234 / 4 | 1.7 | 54.1 (18.1–161.5) | <0.001 |  |
| **Proteinuria ≥ median (≥1550 mg/g)** | 42 / 22 | 52.4 | 124 / 6 | 4.8 | 21.5 (8.1–57.1) | <0.001 | 0.44 |
| **Proteinuria < median** | 42 / 18 | 42.9 | 118 / 3 | 2.5 | 27.0 (7.9–91.8) | <0.001 |  |
| **Creatinine ≥ median (≥1.1 mg/dL)** | 45 / 26 | 57.8 | 132 / 6 | 4.5 | 26.4 (9.9–70.5) | <0.001 | 0.50 |
| **Creatinine < median** | 39 / 14 | 35.9 | 110 / 3 | 2.7 | 20.0 (5.6–71.3) | <0.001 |  |
| **Age ≥ 40 years** | 43 / 21 | 48.8 | 110 / 4 | 3.6 | 25.2 (8.2–77.8) | <0.001 | 0.61 |
| **Age < 40 years** | 41 / 19 | 46.3 | 132 / 3 | 2.3 | 33.4 (9.3–120.5) | <0.001 |  |

**Abbreviations:** RoGSG, ratio of globally sclerotic glomeruli; IS, immunosuppressive therapy; TA/IFTA, tubular atrophy/interstitial fibrosis; OR, odds ratio. *Odds ratios represent the relative odds of composite outcome in the high vs. low RoGSG group within each stratum. Interaction p values derive from logistic models including RoGSG×subgroup term. Given multiple testing and limited events, results are exploratory.*
